# Supplementary material for: Gas therapy potentiates aggregation-induced emission luminogen-based photoimmunotherapy of poorly immunogenic tumors through cGAS-STING pathway activation
Source: Nat Commun. 2023 May 23;14:2950. doi: 10.1038/s41467-023-38601-7 (PMC10205712; doi:10.1038/s41467-023-38601-7)
Supplement: Supplementary file 1 — Supplementary Information [file 41467_2023_38601_MOESM1_ESM.pdf]

Supplementary Information

Gas therapy potentiates aggregation-induced emission luminogen-based photoimmunotherapy of poorly immunogenic tumor through cGAS-STING pathway activation

Wang et al.

Supplementary Figures

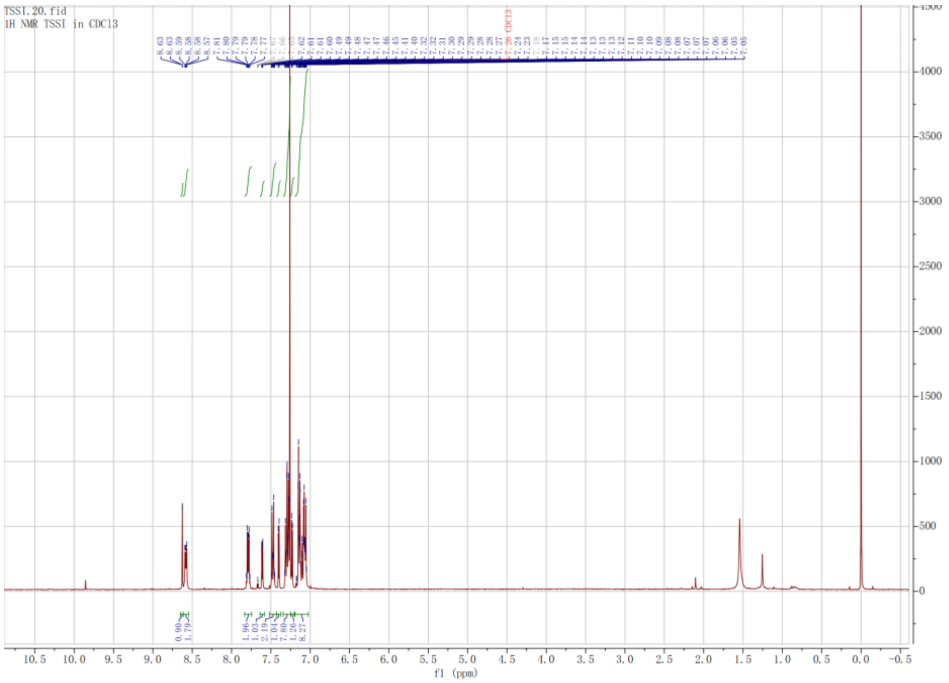

Supplementary Figure 1. ^{1}H NMR spectrum of TSSI.

^{1}H NMR (400 MHz, Chloroform-d) δ 8.65 (d, J = 0.8 Hz, 1H), 8.57 (dd, J = 5.9, 3.2 Hz, 2H), 7.78 (dd, J = 6.0, 3.2 Hz, 2H), 7.65 (dd, J = 4.2, 0.8 Hz, 1H), 7.55-7.49 (m, 2H), 7.38-7.28 (m, 5H), 7.20-7.11 (m, 6H), 7.07-7.01 (m, 2H).

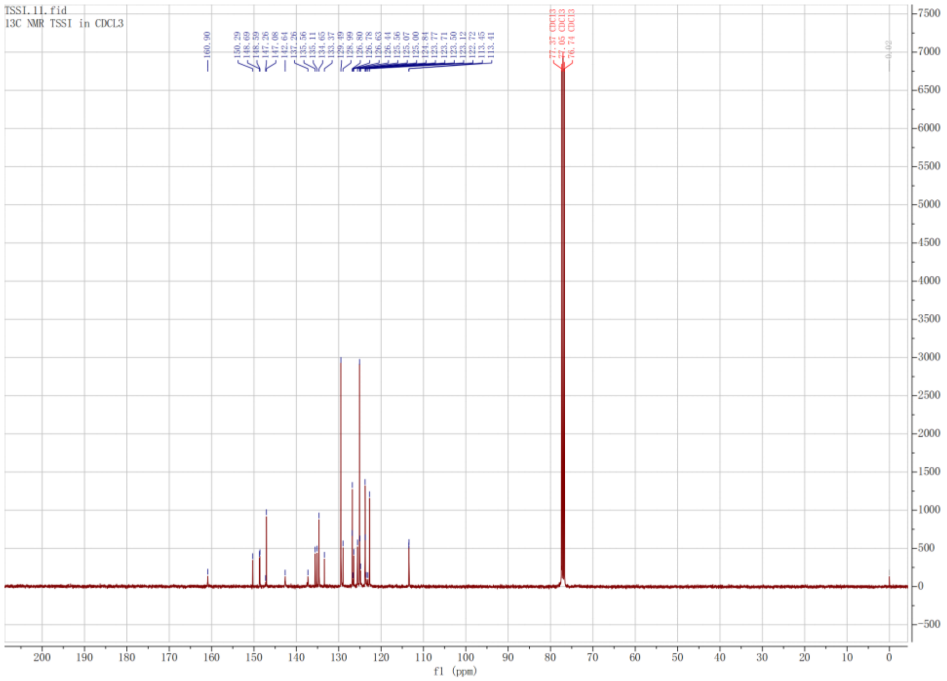

Supplementary Figure 2. ^{13}C NMR spectrum of TSSI.

^{13}C NMR (101 MHz, CDCl3) δ 161.03, 150.43, 148.82, 148.71, 147.20, 142.78, 137.39, 135.70, 135.22, 134.76, 133.50, 129.60, 129.48, 129.11, 126.91, 126.59, 125.68, 125.18, 125.12, 124.77, 123.88, 123.84, 122.86, 113.56, 113.52, 77.48, 77.16, 76.84.

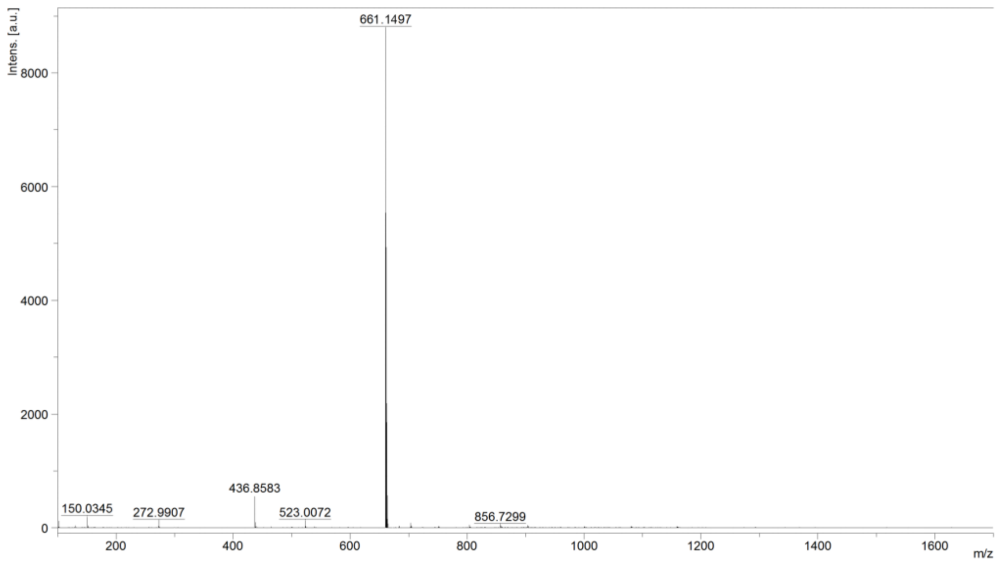

Supplementary Figure 3. HRMS spectrum of TSSI.

HRMS (MALDI-TOF): calcd. for $C_{42}H_{23}N_5S_2$ [M]: 661.1395, found: 661.1497.

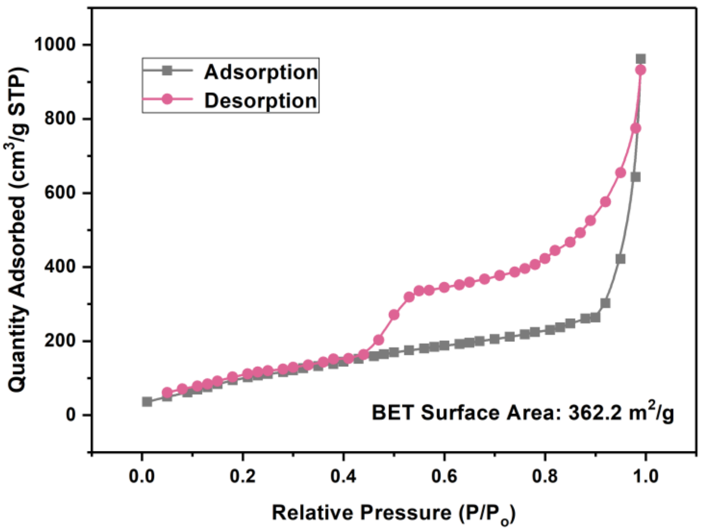

Supplementary Figure 4. Representative nitrogen adsorption-desorption isotherm of tvHMS (*n* = 3 independent experiments). Source data are provided as a Source Data file.

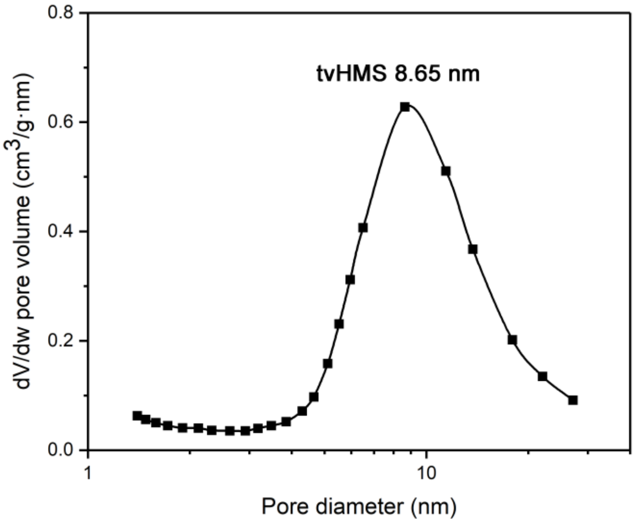

Supplementary Figure 5. Representative measurement of pore diameter distribution of tvHMS (n = 3 independent experiments). Source data are provided as a Source Data file.

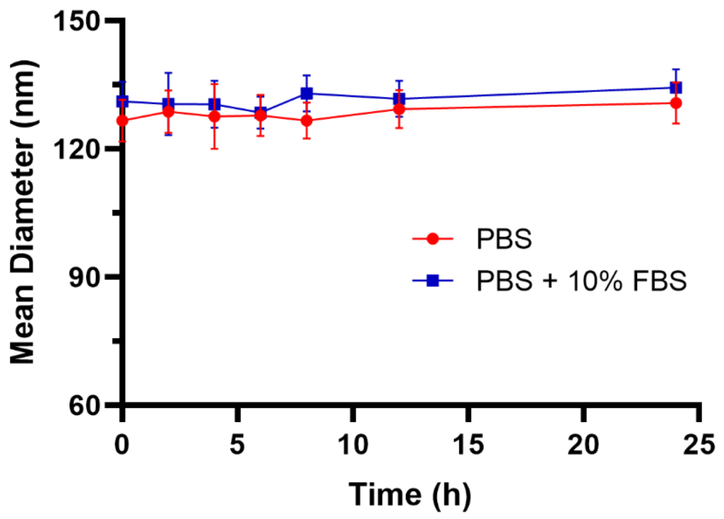

Supplementary Figure 6. The stability of MTHMS in PBS or PBS + 10% FBS (n = 3 independent experiments). Data represent the mean ± SD. Source data are provided as a Source Data file.

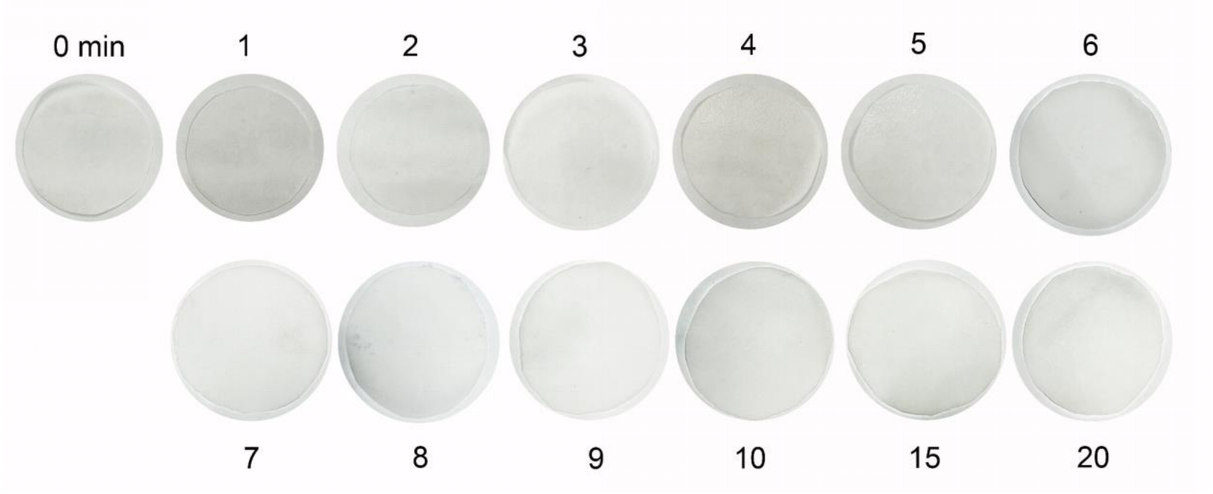

Supplementary Figure 7. Representative detection results of the time-dependent color change of $Pb(NO_3)_2$ test paper to demonstrate the H_2S generation from dvHMS dispersed GSH aqueous solution (*n* = 3 independent experiments).

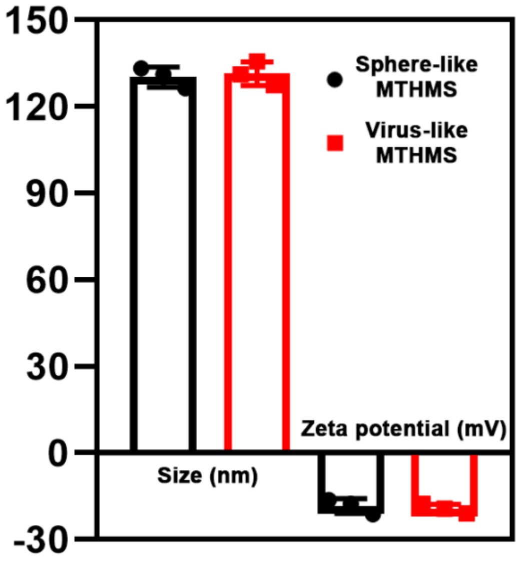

Supplementary Figure 8. Size distribution and zeta potential of sphere-like MTHMS and virus-like MTHMS through dynamic light scattering (DLS) (n = 3 independent experiments). Data represent the mean ± SD. Source data are provided as a Source Data file.

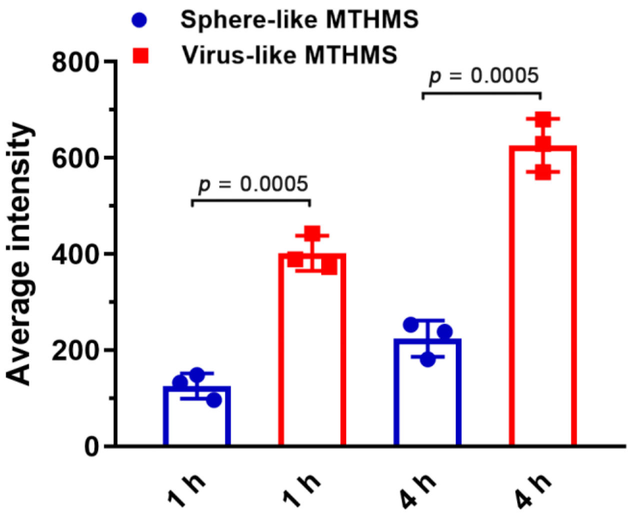

Supplementary Figure 9. Flow cytometric quantification of 4T1 cells incubated with sphere-like MTHMS and virus-like MTHMS for 1 h and 4 h (n = 3 independent experiments). Data represent the mean ± SD. Statistical significance was calculated through two-tailed student's t-test. Source data are provided as a Source Data file.

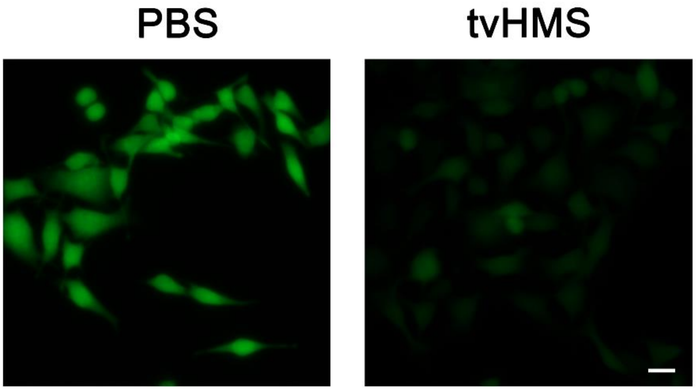

Supplementary Figure 10. ThiolTracker Violet (GSH) fluorescence imaging of 4T1 cancer cells after tvHMS treatment (scale bar = 15 μm). Experiment was repeated three times independently with similar results.

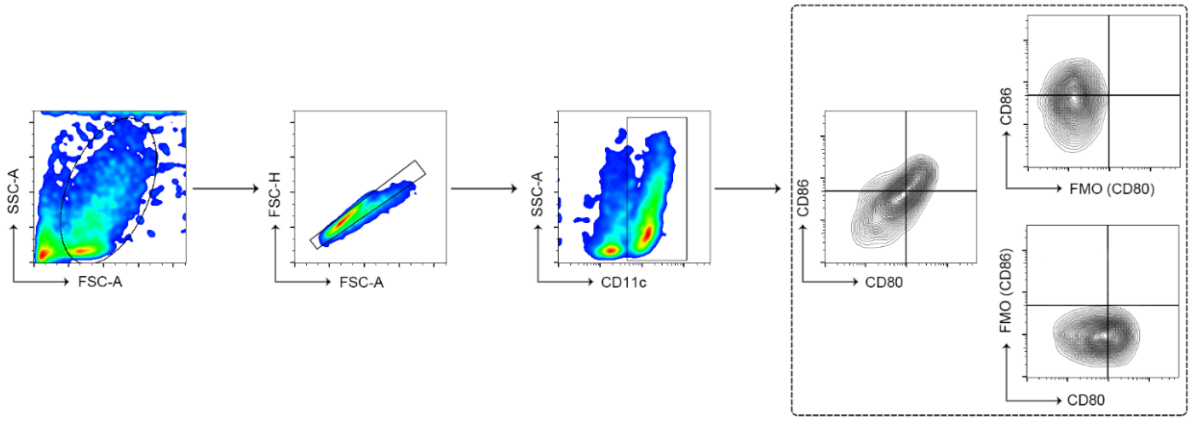

Supplementary Figure 11. Gating strategy and fluorescence minus one (FMO) control for the flow cytometry analysis of DC maturation in vitro (cf. **Figure 4d** and **Supplementary Figure 12a**).

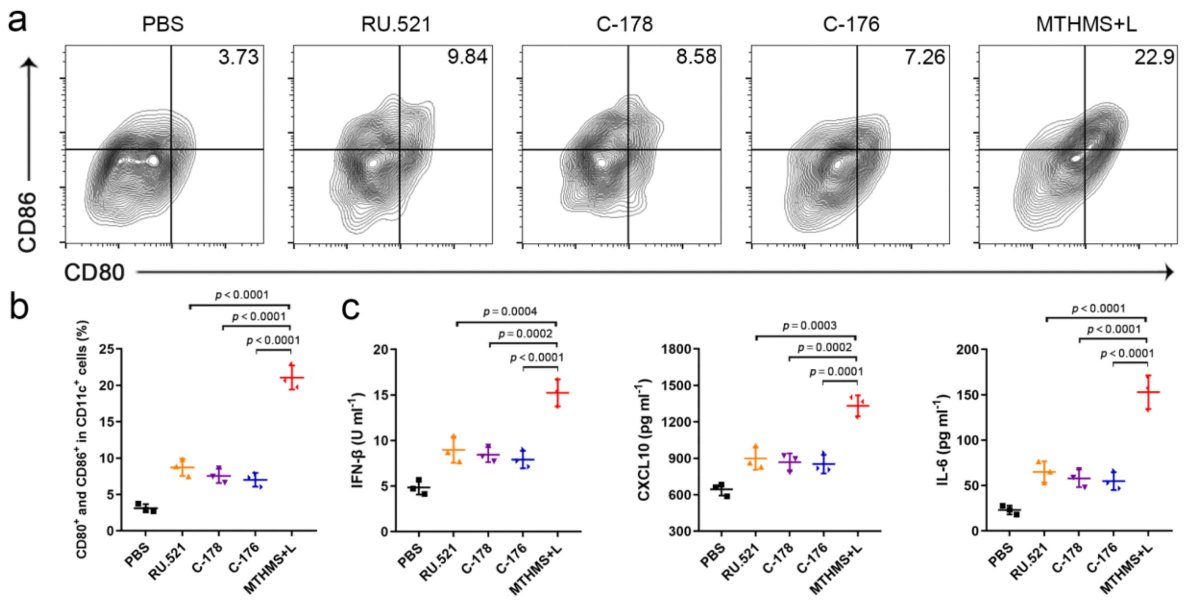

Supplementary Figure 12. The confirmation of cGAS-STING activation-induced DC maturation. (**a**) Flow cytometric assessment images and (**b**) relative quantification of DC maturation (CD11c^{+}CD80^{+}CD86^{+}) triggered by MTHMS+L treated cancer cells with or without cGAS-STING inhibitors (RU.521, C-178, or C-176) (n = 3 independent experiments). For (**b**), the p values of MTHMS+L to C-176, C-178, and Ru.521 are all <0.0001. **c** The detection of cytokines (IFN-β, CXCL10, and IL-6) in culture supernatants of BMDCs incubated with MTHMS+L treated cancer cells with or without RU.521, C-178, or C-176 (n = 3 independent experiments). For (**c**), the p values of MTHMS+L to C-176, C-178, and RU.521 in IFN-β detection are <0.0001, 0.0002, and 0.0004, respectively. The p values of MTHMS+L to C-176, C-178, and RU.521 in CXCL10 detection are 0.0001, 0.0002, and 0.0003, respectively. The p values of MTHMS+L to C-176, C-178, and RU.521 in IL-6 detection are all <0.0001. Data represent the mean ± SD. Statistical significance was calculated through one-way ANOVA using a Tukey post-hoc test. Source data underlying panels **b**,**c** are provided as a Source Data file.

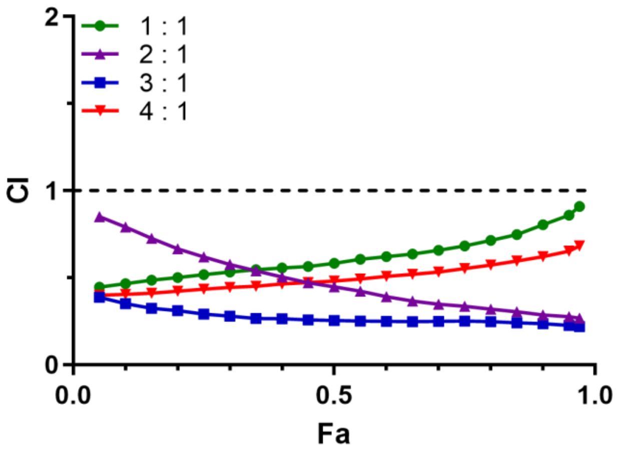

Supplementary Figure 13. Representative CI-Fa curves of MnCO: TSSI in 4T1 cells for 24 h (n = 3 independent experiments). Source data are provided as a Source Data file.

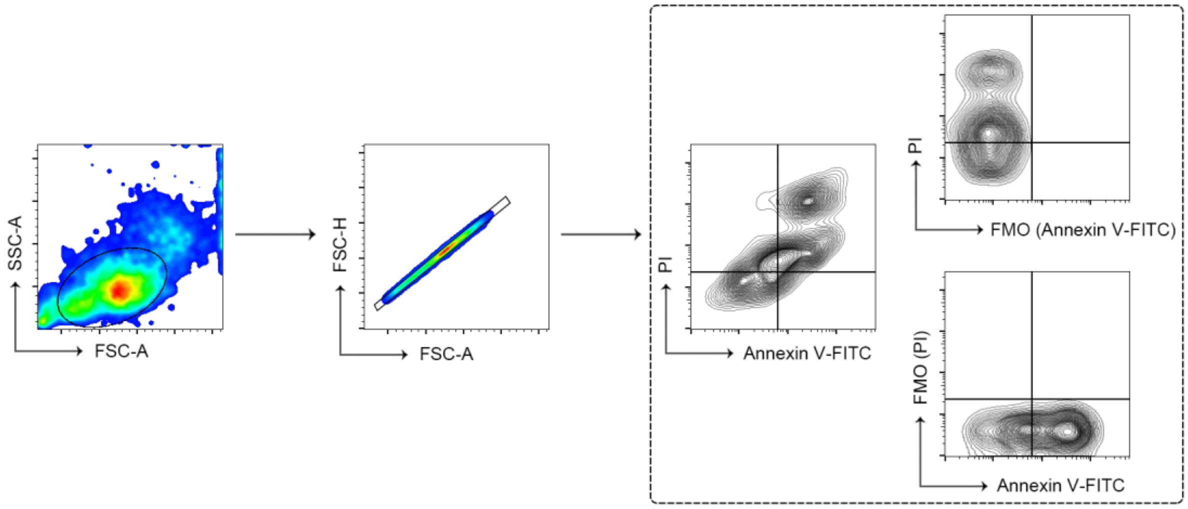

Supplementary Figure 14. Gating strategy and fluorescence minus one (FMO) control for the flow cytometry analysis of apoptosis of 4T1 cancer cells (cf. **Figure 4h** and **Supplementary Figure 15a**).

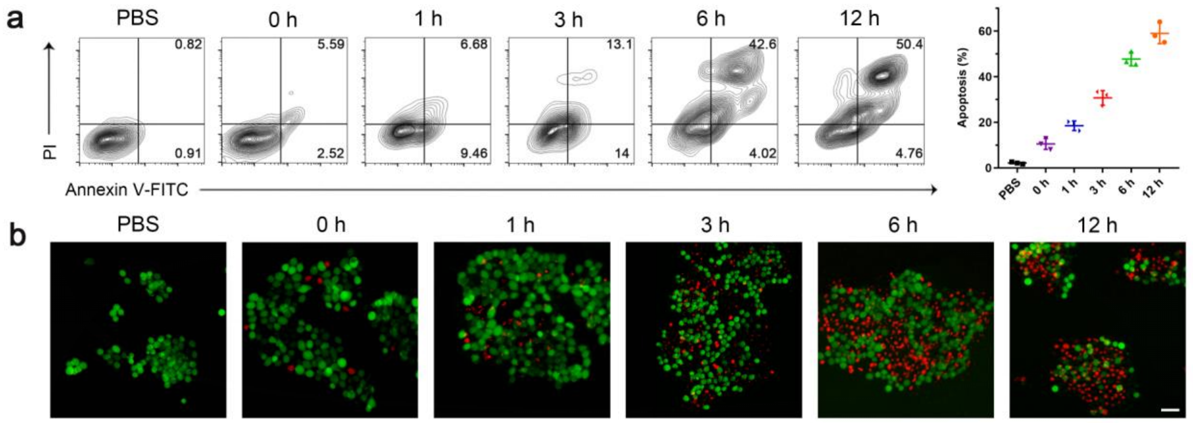

Supplementary Figure 15. A time course for the cell death. **a** Flow cytometric assessment images and relative quantification of apoptosis of 4T1 cancer cells. After incubation with MTHMS for 12 h, cells were then illuminated with 660 nm irradiation (0.3 W cm^{-2}) for 5 min. Following further 0, 1, 3, 6, 12 h incubation, cells were stained with Annexin V-FITC/PI (n = 3 independent experiments). Data represent the mean ± SD. **b** Calcein-AM/PI staining of 4T1 cells at 0, 1, 3, 6, 12 h after laser irradiation (scale bar = 40 μm). Experiment was repeated three times independently with similar results. Source data underlying panel **a** are provided as a Source Data file.

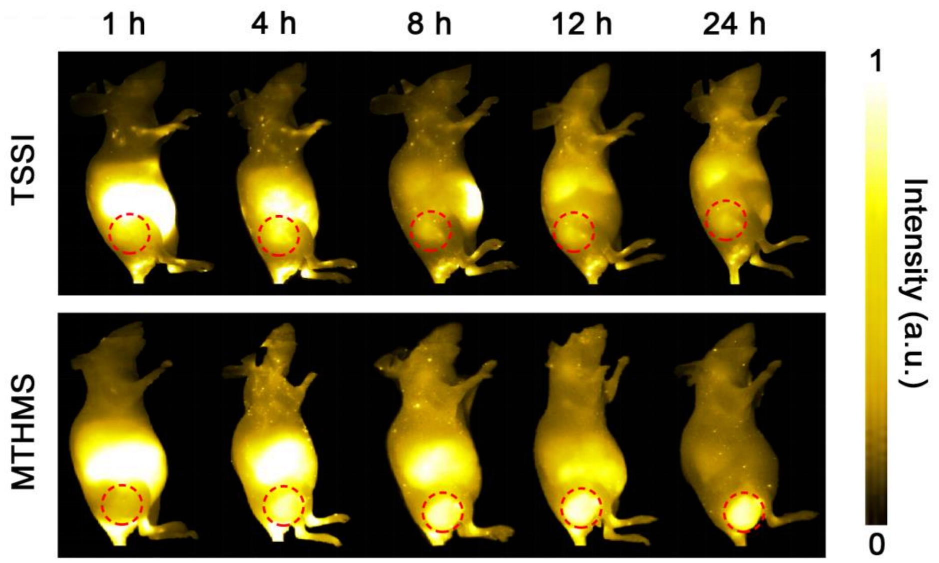

Supplementary Figure 16. Representative NIR-II fluorescence images of tumor-bearing mice at different time points after intravenous administration of free TSSI solution and MTHMS (*n* = 3 mice).

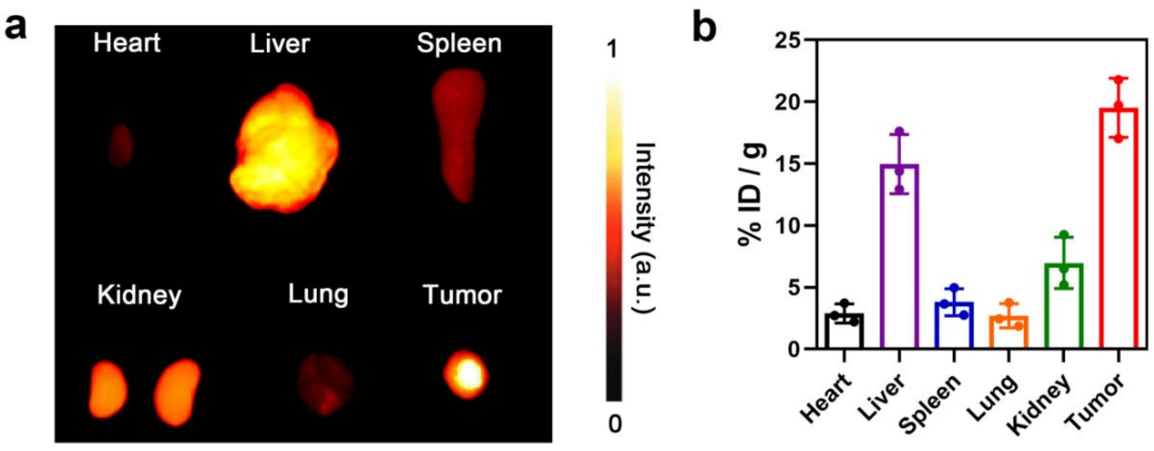

Supplementary Figure 17. Biodistribution of MTHMS. (**a**) *Ex vivo* NIR-II fluorescence images and (**b**) the quantitative biodistribution of tumor and major organs after intravenous injection of MTHMS for 24 h (n = 3 mice). Data represent the mean ± SD. Source data underlying panel **b** are provided as a Source Data file.

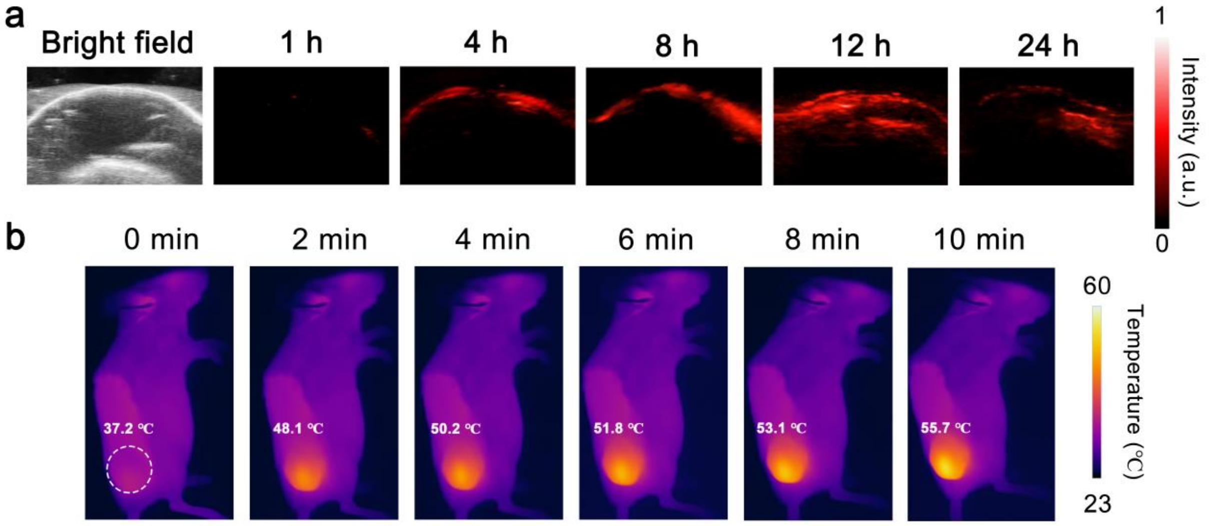

Supplementary Figure 18. Multimodal imaging of MTHMS. **a** Representative photoacoustic images of tumor tissues at different time points after intravenous administration of MTHMS (*n* = 3 mice). **b** Representative thermal images and heating temperatures (at tumor sites) of tumor-bearing mice during continuous NIR irradiation at 12 h post-injection of MTHMS (*n* = 3 mice).

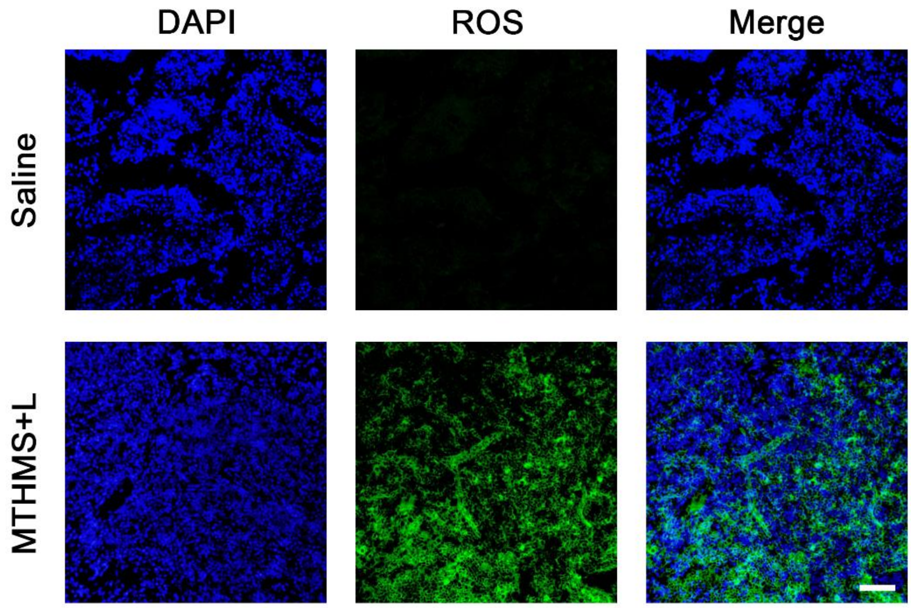

Supplementary Figure 19. Representative images of DCFH-DA (ROS) staining of tumor from mice treated with MTHMS+L (*n* = 3 mice). Scale bar = 100 μm.

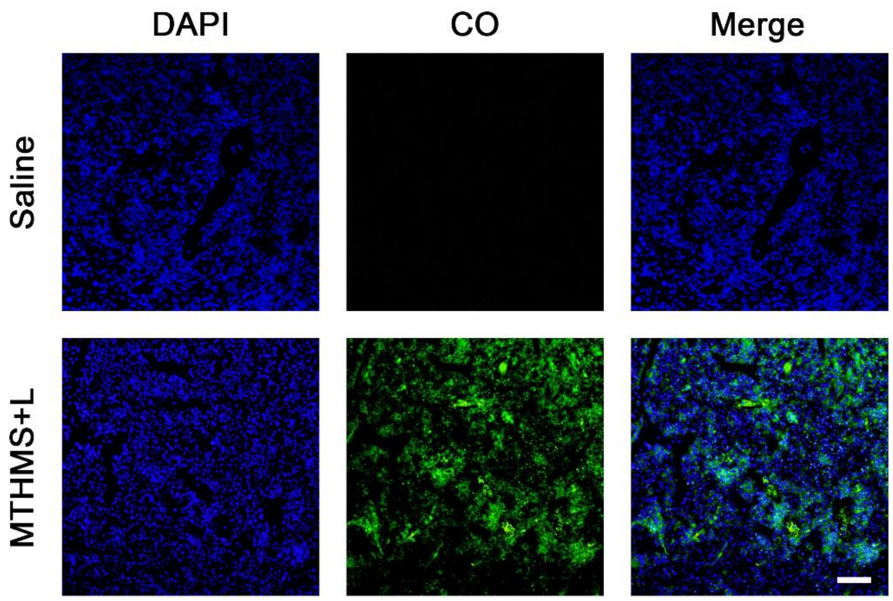

Supplementary Figure 20. Representative images of FL-CO-1 (CO) staining of tumor from mice treated with MTHMS+L (*n* = 3 mice). Scale bar = 100 μm.

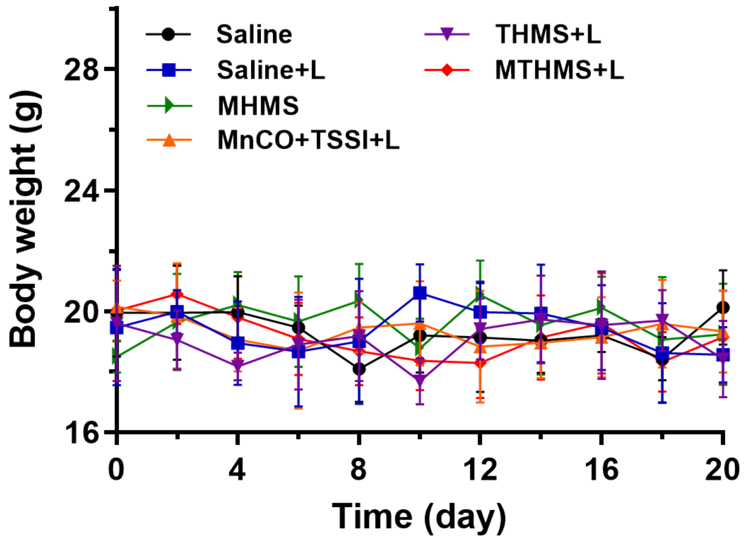

Supplementary Figure 21. Body weight changes after the indicated treatments in 4T1 tumor model (*n* = 5 mice). Data represent the mean ± SD. Source data are provided as a Source Data file.

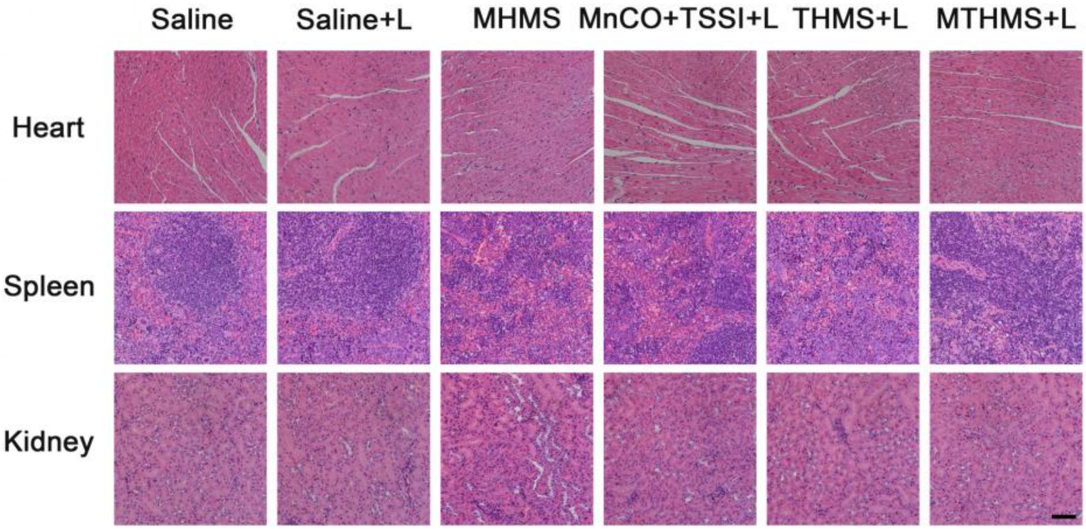

Supplementary Figure 22. Representative images of H&E staining for 4T1 tumor model. H&E staining of heart, spleen, and kidney after various treatments (*n* = 3 mice). Scale bar = 100 μm.

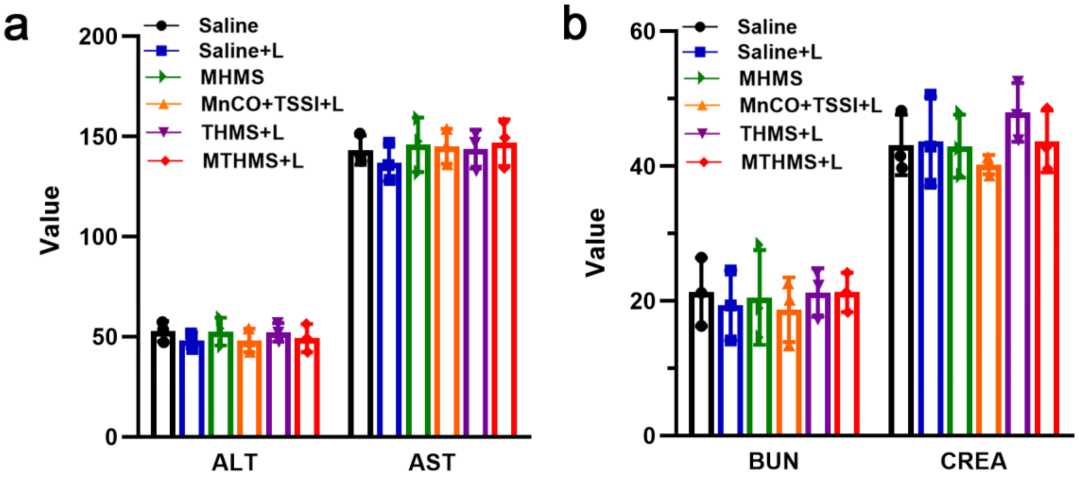

Supplementary Figure 23. Hepatorenal function parameters of 4T1 tumor-bearing mice after intravenous injection of different formulations (*n* = 3 mice). (**a**) ALT (U L^{-1}): alanine aminotransferase; AST (U L^{-1}): aspartate aminotransferase; (**b**) BUN (mmol L^{-1}): blood urea nitrogen; CREA (μmol L^{-1}): creatinine. Data represent the mean ± SD. Source data are provided as a Source Data file.

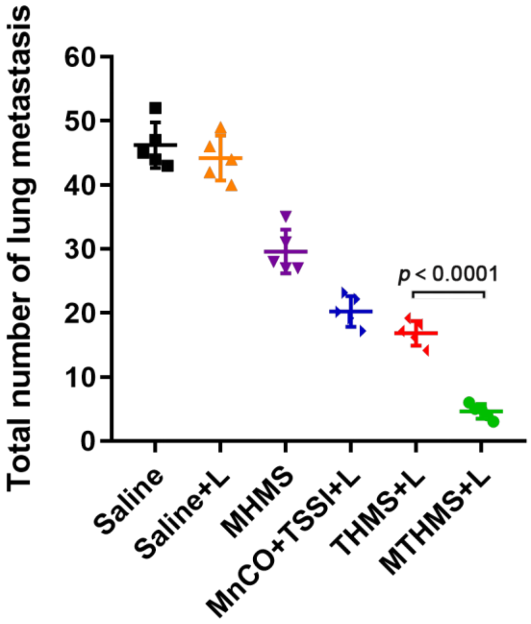

Supplementary Figure 24. The average number of surface lung metastases (*n* = 5 mice). The *p* value of MTHMS+L to THMS+L is <0.0001. Data represent the mean ± SD. Statistical significance was calculated through one-way ANOVA using a Tukey post-hoc test. Source data are provided as a Source Data file.

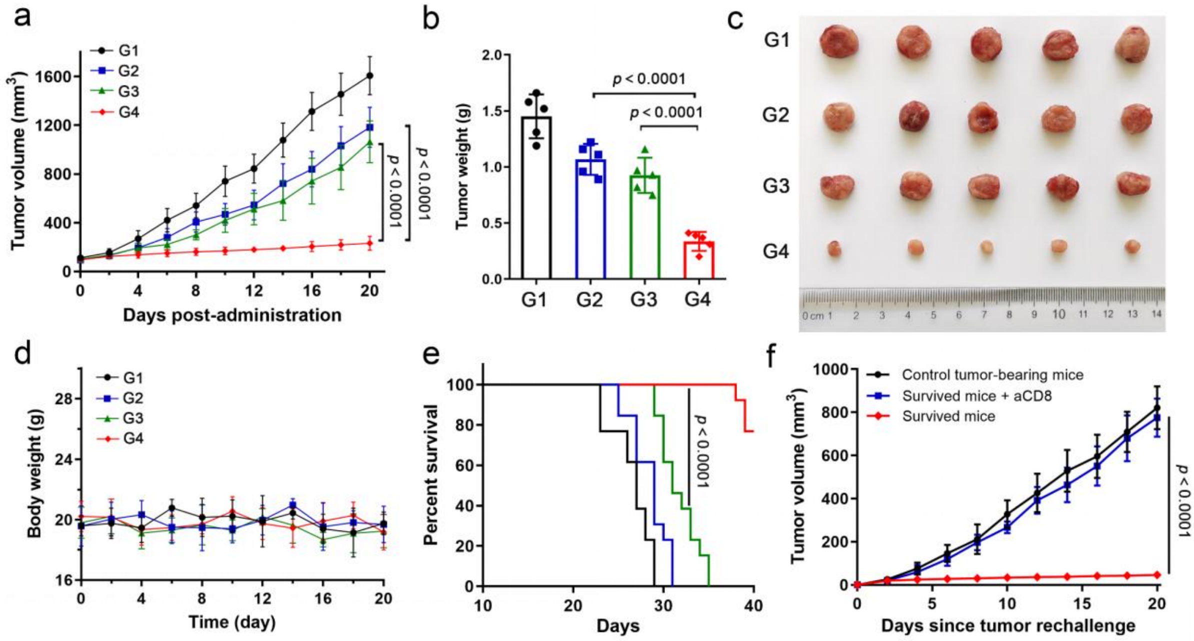

Supplementary Figure 25. Assessment of therapeutic efficiency of MTHMS (G1: Saline, G2: MTHMS+L+aCD8, G3: MTHMS, G4: MTHMS+L). (**a**) Tumor growth curve (n = 5 mice), (**b**) tumor weight variations (n = 5 mice), (**c**) tumor photographs (n = 5 mice), (**d**) body weight changes (n = 5 mice) and (**e**) survival curve (n = 13 mice) following different treatments. For (**a-b**), the p values of G4 to G3 and G4 to G2 are all <0.0001. For (**e**), the p value of G4 to G3 is <0.0001. **f** Tumor growth curve of mice rechallenged with 4T1 cancer cells (n = 5 mice). For (**f**), the p value of Survived mice to Survived mice+aCD8 is <0.0001. Data represent the mean ± SD. Statistical significance was calculated through one-way ANOVA using a Tukey post-hoc test (**a**, **b**, **f**) or log-rank (Mantel-Cox) test (**e**). Source data underlying panels **a**,**b**,**d-f** are provided as a Source Data file.

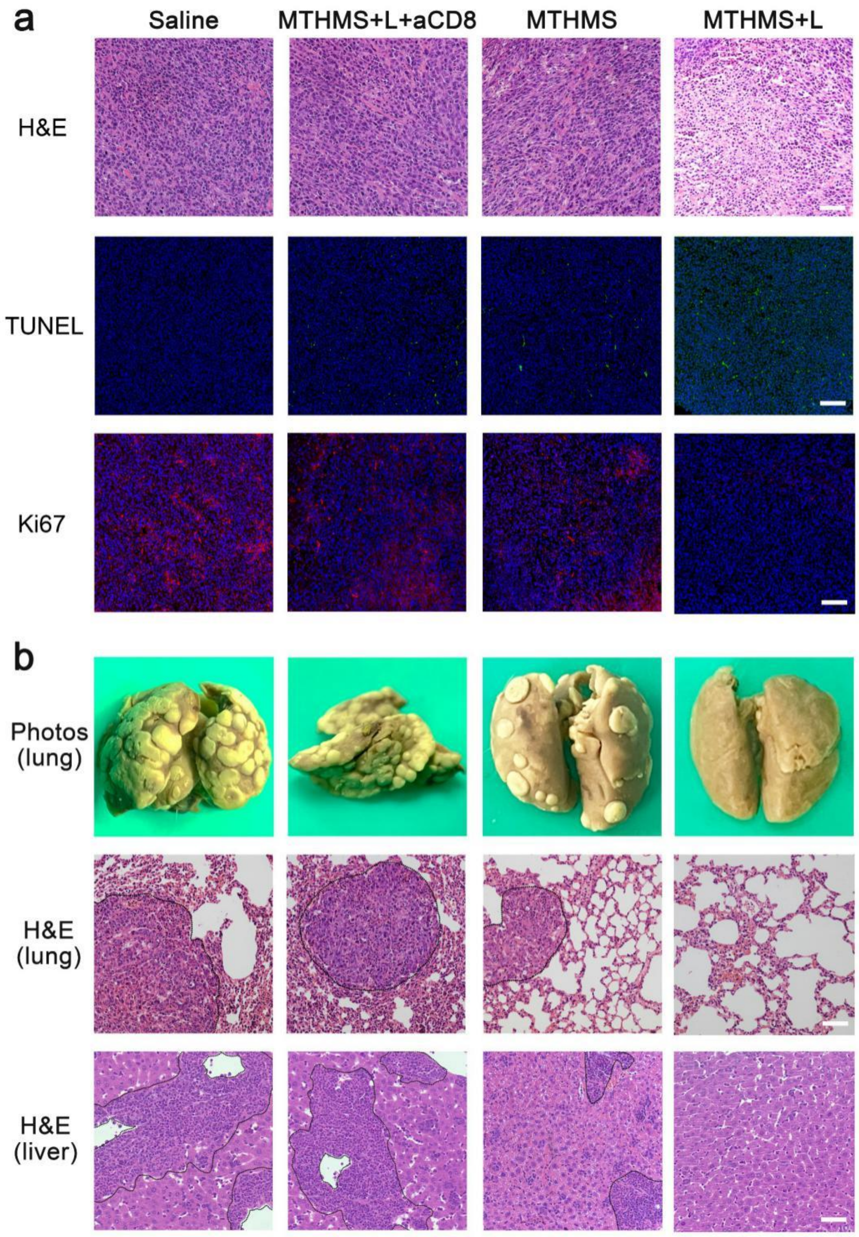

Supplementary Figure 26. Assessment of therapeutic efficiency of MTHMS. **a** Representative images of H&E, TUNEL, and Ki67 staining of tumor slices collected from mice receiving various treatments (n = 3 mice). **b** Representative photos of lung stained with Bouin's fluid and representative images of H&E staining of lung and liver following different treatments (n = 3 mice). Dashed outlines indicate lung and liver metastases in H&E staining. Scale bar = 100 µm.

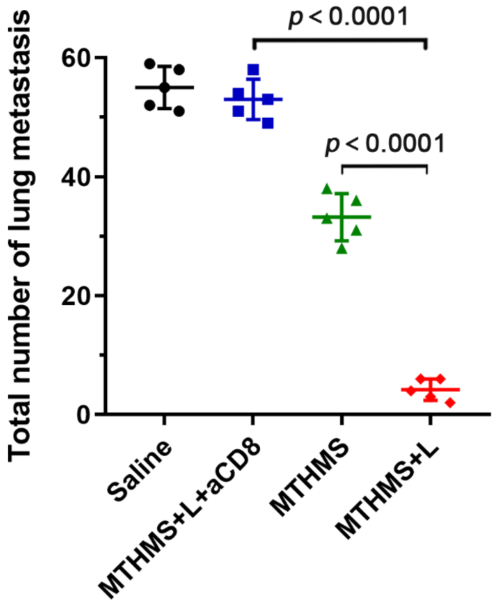

Supplementary Figure 27. The average number of surface lung metastases (*n* = 5 mice). The *p* values of MTHMS+L to MTHMS and MTHMS+L to MTHMS+L+aCD8 are both <0.0001. Data represent the mean ± SD. Statistical significance was calculated through one-way ANOVA using a Tukey post-hoc test. Source data are provided as a Source Data file.

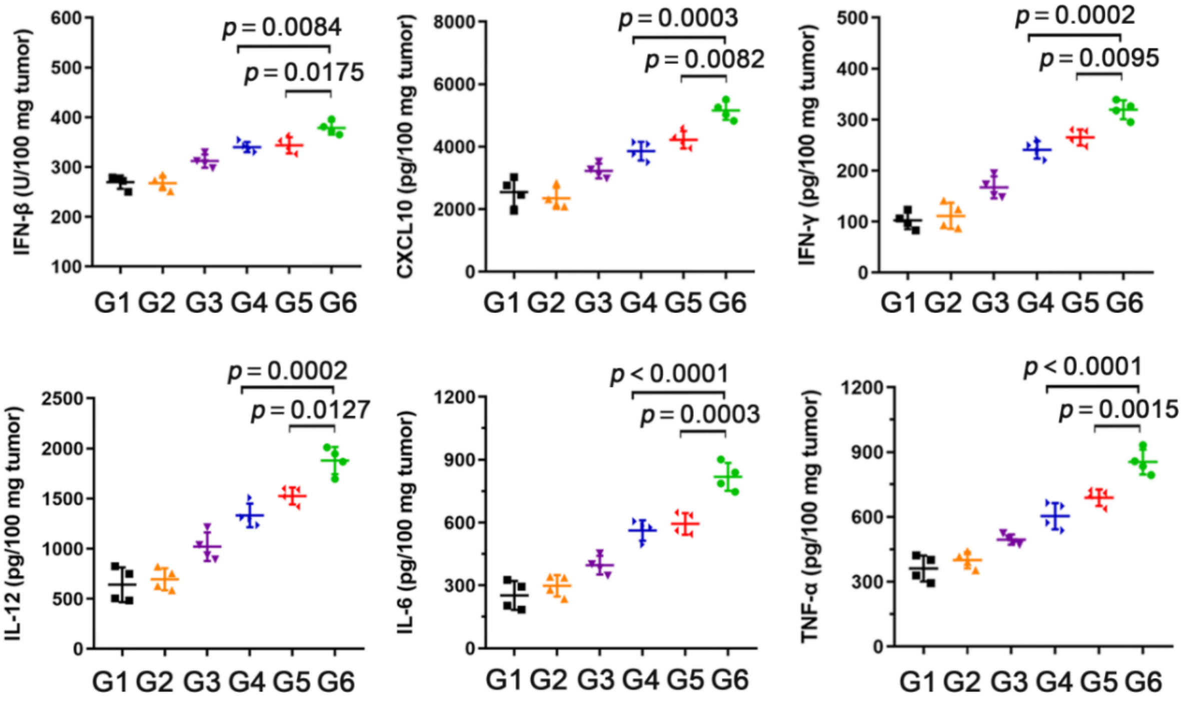

Supplementary Figure 28. The secretion of cGAS-STING-related cytokines (IFN-β, CXCL10) and proinflammatory cytokines (IFN-γ, IL-12, IL-6, TNF-α) of tumor supernatant in 4T1 tumor model ($n = 4$ mice). G1: Saline, G2: Saline+L, G3: MHMS, G4: MnCO+TSSI+L, G5: THMS+L, G6: MTHMS+L. The p values of G6 to G5 and G6 to G4 in IFN-β secretion are 0.0175 and 0.0084, respectively. The p values of G6 to G5 and G6 to G4 in CXCL10 secretion are 0.0082 and 0.0003, respectively. The p values of G6 to G5 and G6 to G4 in IFN-γ secretion are 0.0095 and 0.0002, respectively. The p values of G6 to G5 and G6 to G4 in IL-12 secretion are 0.0127 and 0.0002, respectively. The p values of G6 to G5 and G6 to G4 in IL-6 secretion are 0.0003 and <0.0001, respectively. The p values of G6 to G5 and G6 to G4 in TNF-α secretion are 0.0015 and <0.0001, respectively. Data represent the mean ± SD. Statistical significance was calculated through one-way ANOVA using a Tukey post-hoc test. Source data are provided as a Source Data file.

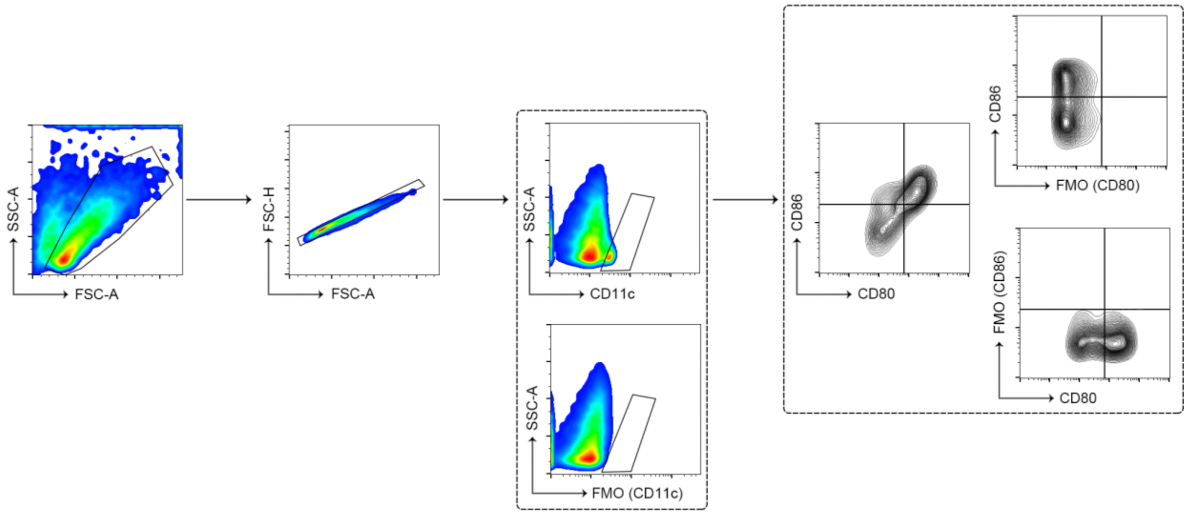

Supplementary Figure 29. Gating strategy and fluorescence minus one (FMO) control for the flow cytometry analysis of DC maturation in TDLNs (cf. **Figure 6c**).

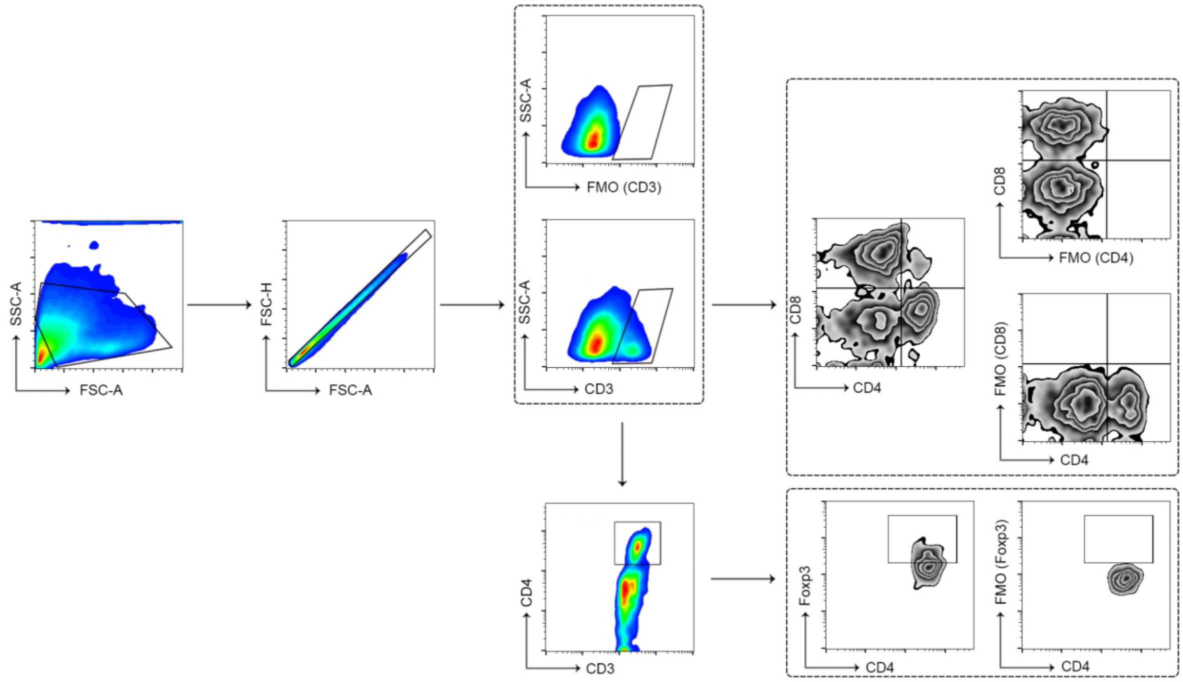

Supplementary Figure 30. Gating strategy and fluorescence minus one (FMO) control for the flow cytometry analysis of tumor-infiltrating CD8$^+$ T cells and CD4$^+$Foxp3$^+$ Tregs (cf. **Figure 6d**, **e**).

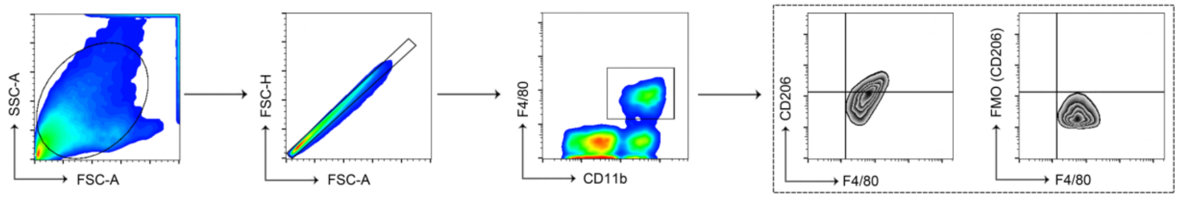

Supplementary Figure 31. Gating strategy and fluorescence minus one (FMO) control for the flow cytometry analysis of M2-like macrophages (CD206^{hi}CD11b^{+}F4/80^{+}) in tumor (cf. **Figure 6f**).

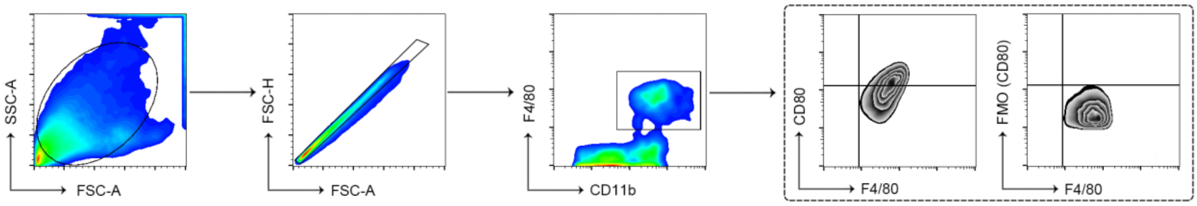

Supplementary Figure 32. Gating strategy and fluorescence minus one (FMO) control for the flow cytometry analysis of M1-like macrophages ($CD80^{hi}CD11b^{+}F4/80^{+}$) in tumor (cf. **Figure 6g**).

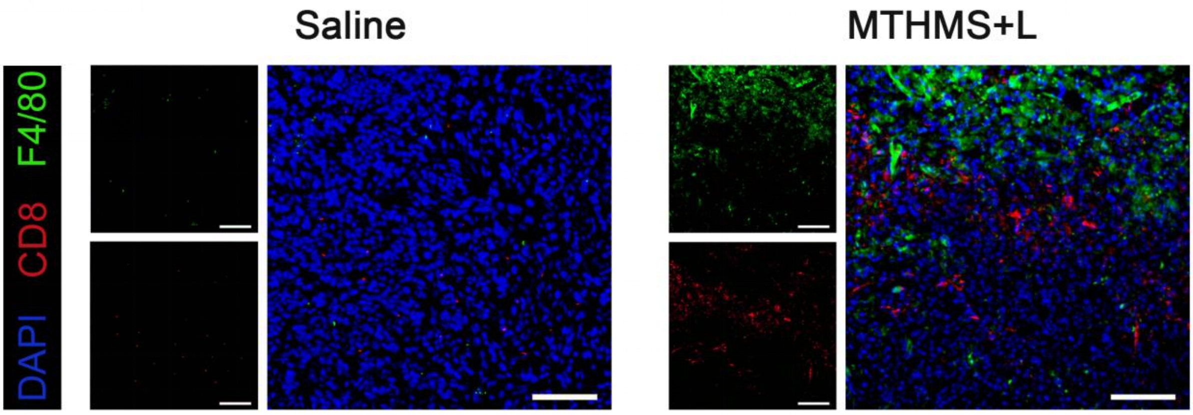

Supplementary Figure 33. Representative immunofluorescence images of tumors in 4T1 tumor model. Immunofluorescence images of tumors displaying $CD8^+$ T cell and $F4/80^+$ macrophage infiltration for saline and MTHMS+L groups (*n* = 3 mice). Scale bar = 50 μm.

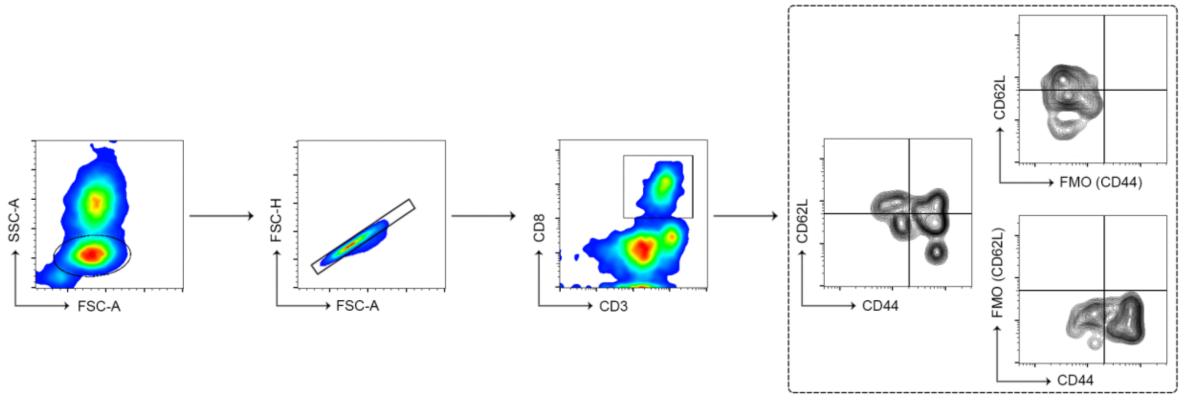

Supplementary Figure 34. Gating strategy and fluorescence minus one (FMO) control for the flow cytometry analysis of $CD3^{+}CD8^{+}CD62L^{low}CD44^{hi}$ T_{EM} in spleen (cf. **Figure 6h**).

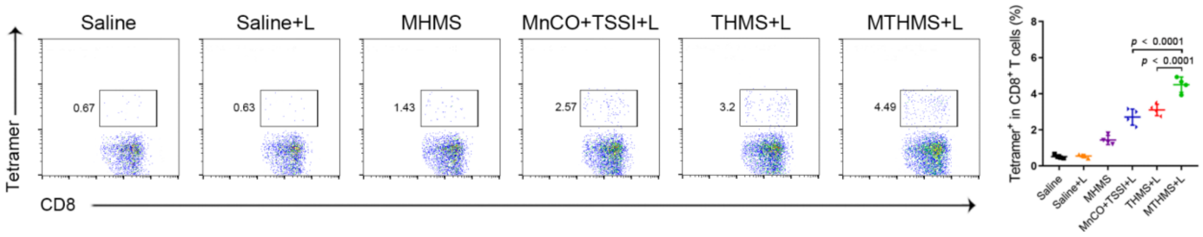

Supplementary Figure 35. Flow cytometric assay and relative quantification of gp70 tetramer staining of CD8$^+$ T cells in spleen in 4T1 tumor model (*n* = 4 mice). The *p* values of MTHMS+L to THMS+L and MTHMS+L to MnCO+TSSI+L are both <0.0001. Data represent the mean ± SD. Statistical significance was calculated through one-way ANOVA using a Tukey post-hoc test. Source data are provided as a Source Data file.

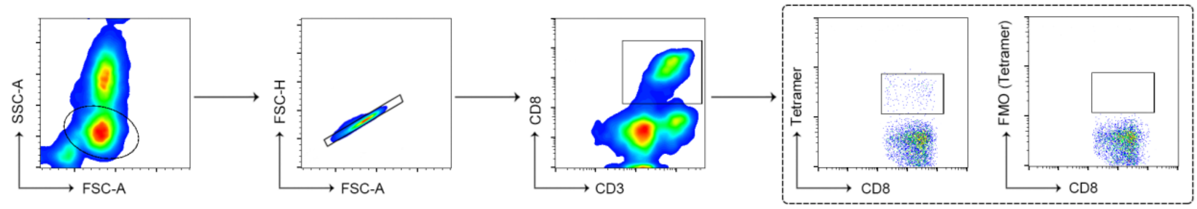

Supplementary Figure 36. Gating strategy and fluorescence minus one (FMO) control for the flow cytometry analysis of gp70 tetramer specific CD8^{+} T cells in spleen (cf. **Supplementary Figure 35**, **38**).

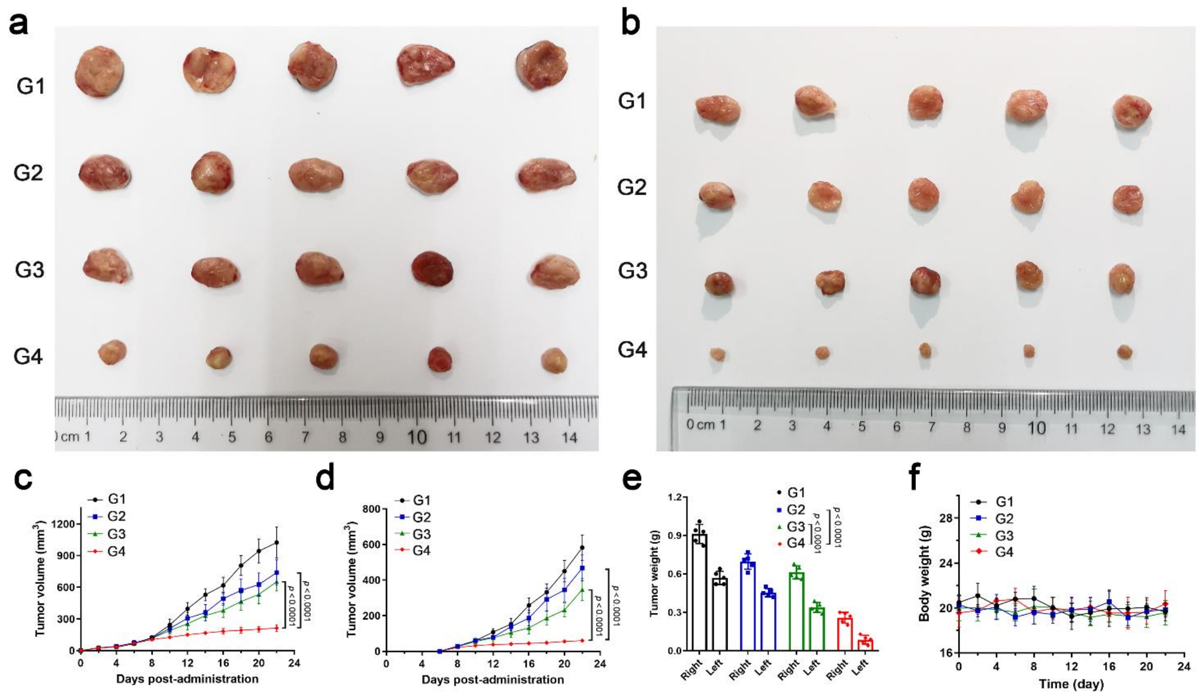

Supplementary Figure 37. Assessment of therapeutic efficiency of MTHMS in abscopal model (G1: Saline+L, G2: MTHMS+L+aCD8, G3: MTHMS, G4: MTHMS+L). Tumor photographs of (**a**) primary tumor (right) and (**b**) distant tumor (left). Tumor growth profiles of (**c**) primary tumor (right) and (**d**) distant tumor (left). (**e**) Tumor weight variations and (**f**) body weight changes after the indicated treatments (n = 5 mice). For (**c-e**), the p values of G4 to G3 and G4 to G2 are both <0.0001. Data represent the mean ± SD. Statistical significance was calculated through one-way ANOVA using a Tukey post-hoc test. Source data underlying panels **c-f** are provided as a Source Data file.

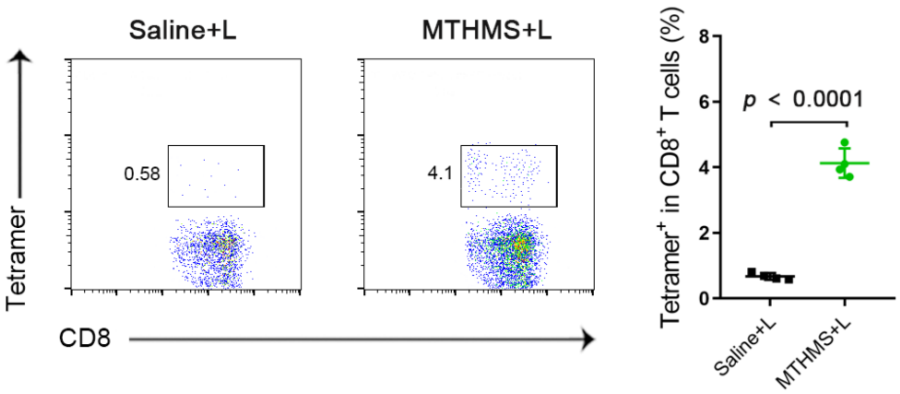

Supplementary Figure 38. Flow cytometric assay and relative quantification of gp70 tetramer staining of $CD8^+$ T cells in spleen in the bilateral tumor model (n = 4 mice). The p value of MTHMS+L to Saline+L is <0.0001. Data represent the mean ± SD. Statistical significance was calculated through two-tailed student's t-test. Source data are provided as a Source Data file.

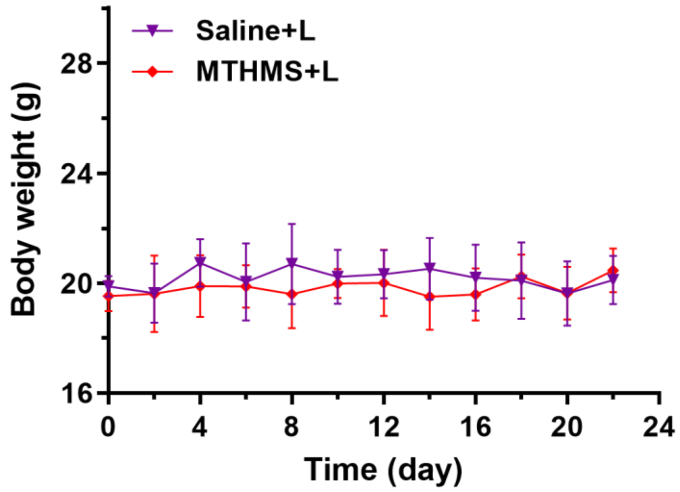

Supplementary Figure 39. Body weight changes after the indicated treatments in bilateral tumor model (n = 5 mice). Data represent the mean ± SD. Source data are provided as a Source Data file.

Supplementary Tables

Supplementary Table 1. The full names of different formulations.

Formulations	Full names
dvHMS	disulfide bond incorporated virus-like hollow mesoporous silica
tsHMS	tetrasulfide-functionalized sphere-like hollow mesoporous silica
tvHMS	tetrasulfide-functionalized virus-like hollow mesoporous silica
MHMS	manganese carbonyl (MnCO) encapsulated tvHMS
THMS	TSSI encapsulated tvHMS
MTHMS	MnCO and TSSI co-encapsulated tvHMS

Supplementary Table 2. Cytotoxicity (IC_{50} values[a)]) of MHMS, MnCO+TSSI+L, THMS+L, and MTHMS+L to 4T1 cells (CCK-8 assay).

Formulations	4T1 (μg mL^{-1})	
	TSSI	MnCO
MHMS	-	139.3
MnCO+TSSI+L	14.11	42.33
THMS+L	10.45	-
MTHMS+L	2.735	8.204

[a)] Half maximal inhibitory concentration, presented as equivalent concentrations of TSSI and MnCO.
